# *Drosophila* Embryos as Model to Assess Cellular and Developmental Toxicity of Multi-Walled Carbon Nanotubes (MWCNT) in Living Organisms

**DOI:** 10.1371/journal.pone.0088681

**Published:** 2014-02-18

**Authors:** Boyin Liu, Eva M. Campo, Torsten Bossing

**Affiliations:** 1 School of Biological Sciences, University of Bangor, Bangor, United Kingdom; 2 School of Electronic Engineering, University of Bangor, Bangor, United Kingdom; National Cancer Institute, United States of America

## Abstract

Different toxicity tests for carbon nanotubes (CNT) have been developed to assess their impact on human health and on aquatic and terrestrial animal and plant life. We present a new model, the fruit fly *Drosophila* embryo offering the opportunity for rapid, inexpensive and detailed analysis of CNTs toxicity during embryonic development. We show that injected DiI labelled multi-walled carbon nanotubes (MWCNTs) become incorporated into cells in early *Drosophila* embryos, allowing the study of the consequences of cellular uptake of CNTs on cell communication, tissue and organ formation in living embryos. Fluorescently labelled subcellular structures showed that MWCNTs remained cytoplasmic and were excluded from the nucleus. Analysis of developing ectodermal and neural stem cells in MWCNTs injected embryos revealed normal division patterns and differentiation capacity. However, an increase in cell death of ectodermal but not of neural stem cells was observed, indicating stem cell-specific vulnerability to MWCNT exposure. The ease of CNT embryo injections, the possibility of detailed morphological and genomic analysis and the low costs make *Drosophila* embryos a system of choice to assess potential developmental and cellular effects of CNTs and test their use in future CNT based new therapies including drug delivery.

## Introduction

The first report of the synthesis of carbon nanotubes (CNTs) two decades ago [1] sparked interest in such diverse fields as electronics, optics, physics, material sciences, medicine and biology. The promise CNTs hold for these fields originates from their unique physical, chemical, electrical and mechanical properties [2]. Consequently, commercial production and applications are increasing and CNTS have a growing presence in our daily lives ([3], see also Woodrow Wilson Nano Inventory). Accumulation of nanoparticles in our environment is still at the detection threshold but the continuous release of particles by production, wear and tear, and waste disposal makes an increased environmental exposure inevitable [4]. In addition, the future use of CNTs in medical applications such as drug delivery, biosensors and surgical scaffolds [5] will increase human contact with CNTs and justifies international efforts for the development and standardisation of existing toxicity tests, as well as of new approaches to test the health impact of CNTs [6].

Environmental concerns and the hazard to human health associated with CNTs have attracted widespread attention [7], [8]. CNTs can cause cellular and tissue damage by stimulating inflammation and necrosis due to increased production of reactive oxygen species (ROS) [7], [9]. Single walled CNTs tend to be more damaging than multi-walled CNTs (MWCNTs) [10]. The shape, length and the addition of side groups also influence CNT toxicity [7], An increasing numbers of studies indicate that many of the toxic effects initially reported may be caused by contaminations deposited during CNT production, an observation explaining some of the inconsistencies in previous studies [7], [9]. Cell cultures are often the medium of choice for toxicity tests since they offer a fast, low cost and high-throughput approach. Yet, cell culture results vary with cell type and culture conditions [9], and results may not translate directly into the whole organism environment where, in a temporal and spatially controlled fashion, thousands of endogenous proteins and hundreds of different cell types interact with each other. Due to high costs, high throughput toxicity studies on mammals are scarce. It may be advantageous to opt for an alternative way, conducting high throughput studies in lower vertebrates and invertebrates with short generation time and high fecundity, and validate results obtained in these studies in a limited number of rodents. Indeed, zebrafish [11], [12], [13] and the flatworm *C.elegans* [14], [15] have been recently used to study the toxicity of CNTs. Both model organisms allow the establishment of basic mechanisms of CNT toxicity by examining viability, fertility, tissue and cellular integrity [16]. They also give an insight into alterations in gene expression changes, which underlie altered organ function [11], [15].

Here we present a third simple animal model system towards the study of CNT toxicity, *Drosophila* embryos. Embryos of *Drosophila* do not only offer all the advantages of zebrafish and *C.elegans* but also have unique features, which allow an unprecedented insight into the mechanisms of cellular toxicity. First, *Drosophila* is widely used to understand the biology of human diseases and also as a tool for gene and drug discovery [17]. Second, during early *Drosophila* embryogenesis, all nuclei share the same cytoplasm, which allows CNTs injected into the egg to interact with nuclei and to become included into the cells forming about 3 hours into embryogenesis. Injections overcome the problem of reduced bioavailability of CNTs and permit the study of CNT effects on cell division, cell survival, organ formation and function after cellular uptake, a likely occurrence during drug delivery or scaffold insertions upon surgery in humans [5]. Third, a plethora of available transgenic flies in which the expression of fluorescently tagged proteins outlines cellular substructures or distinct cell types can be used to study intra- and extracellular distribution of CNTs in living developing embryos [17], [18]. Finally, embryogenesis in *Drosophila* is a well-documented process, right down to the single cell level [19], [20], [21]. Hence any disturbances caused by the presence of CNT in cell movements or cell communication essential for organ formation or cellular differentiation can be easily detected.

In this report we used different subcellular markers to follow the distribution of MWCNTs in living, developing *Drosophila* embryos at a single cell resolution. We show that the intra- and extracellular accumulation of MWCNTs does not interfere with nuclear or cellular divisions or the overall embryonic development. Interestingly also the amount of DNA double-strand breaks known to contribute to genotoxic stress and cancer [22] are not significantly increased, However, MWCNTs induced a decrease in the survival of ectodermal stem cells, which was not observed in neural stem cells, suggesting that stem cell types differ in their vulnerability to MWCNTs exposure. Our study indicates that *Drosophila* can be used as a tool to study the toxicity of MWCNTs. The availability of near unlimited numbers of embryos, the ease of embryo injections and the low cost of the procedure make *Drosophila* embryos a model of choice to study developmental toxicity of CNTs in whole organisms.

## Results

### Injected MWCNTs are incorporated into cells and do not disrupt embryonic development or cell motility

We sought to test the toxicity of MWCNTs for cell division, cellular differentiation and overall embryonic development by injecting MWCNTs into *Drosophila* embryos. In order to visualize live nuclear or cellular divisions, and subsequent organ formation, we used transgenic fly strains. either expressing histones coupled to Yellow Fluorescent Protein (YFP) labeling nuclei/chromosomes or Green Fluorescent Protein (GFP) trapped into the intron of the microtubule-binding protein Jupiter [23], labelling the outer cell membranes and mitotic spindles.

**Figure 1:**
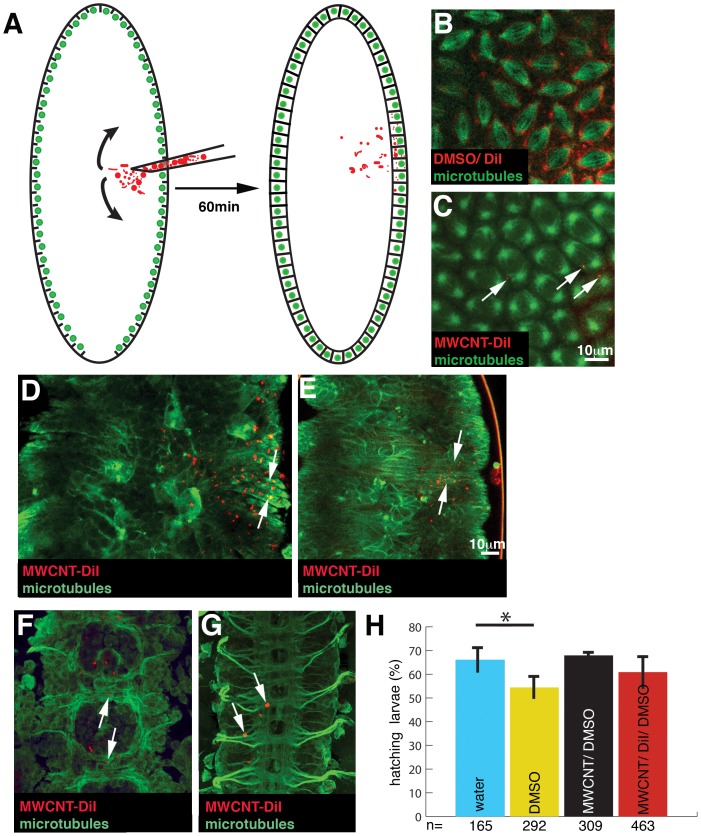
MWCNTs do not interfere with gross embryonic development and viability. Ventral views; Anterior is up. Bar, 10 µm. (A) Using a microcapillary, we injected DiI labelled MWCNTs (red) into the syncytial blastoderm of *Drosophila* embryos. At this stage of development, all nuclei (green) share the same cytoplasm permitting an unhindered diffusion (arrows) of the MWCNTs to nuclei adjacent to the injection site. In the next hour of embryogenesis, invaginating cell membranes will partition the nuclei into single cells incorporating the MWCNTs. (B, C) Live snapshots of embryos injected with 1 mg/ml DiI in DMSO (red, B, DMSO/DiI) or 1 mg/ml DiI labelled MWCNTs in DMSO (red, C, MWCNT-DiI) immediately after injection into an embryo with microtubules labelled by GFP (green, GFP-jupiter). MWCNTs remain in the cytoplasm, the darker area surrounding the microtubules. Note that DiI not bound to MWCNT shows a diffuse membrane stain, whereas DiI bound to MWCNTs can be detected as small puncta (arrow). (D, E) Live confocal section of the same embryo 8 h (D) and 15 h (E) after injection. Cell outlines are labelled by GFP labelled microtubules (green, Jupiter-GFP). The majority of MWCNT (red) has been incorporated into the newly formed epidermis cells (arrows). (F, G) Live confocal section of the developing CNS of the same embryo 8 h (F) and 15 h (G) after injection. Cell outline and axons are labelled by GFP labelled microtubules (green, Jupiter-GFP). MWCNTs do not interfere with the initial outgrowth of axons (F, arrows) and are incorporated into the axonal scaffold without visible damage (G, arrows). (H) MWCNT injections do not interfere with embryonic viability. If embryos develop into healthy larvae, the larvae will hatch out of the egg shell. Hatching rate of embryos injected with water (blue, injection control), 10% DMSO (yellow, vehicle control), 1 mg/ml MWCNT in 10% DMSO/water (black, MWCNT/DMSO, see also Experimental Section) and 1 mg/ml MWCNT in 10% DMSO labelled with DiI (red, MWCNT/DiI/DMSO). Pairwise comparison with water injected embryos (t-Test), *, significant (p<0.05); error bars, StDev;. Y-axis, Hatching rate in % of injected embryos; X-axis, Number of injected embryos (n).

*Drosophila* embryogenesis starts with the syncytial blastoderm when embryos only consist of nuclei, which undergo 13 near synchronous divisions. Before the last 4 divisions, nuclei align along the embryo surface. After the last nuclear division, an actin-based movement results in the ingression of cell membranes from the egg membrane, and each nuclei and its surrounding cytoplasm is partitioned into a newly formed cell [24]. We injected MWCNTs and vehicle controls at the time when nuclei reach the embryonic periphery (Figure 1A). MWCNTs were marked with the red fluorescent lipophilic dye DiI (Invitrogen) as this dye has been shown to bind non-covalently to carbon nanotubes [25] and to be harmless for *Drosophila* embryogenesis [21]. MWCNTs were labelled at 1 mg/ml DiI in 100% DMSO. DiI in 100% DMSO forms a homogenous solution, which does not precipitate when spun at room temperature at 6000 rpm. However, addition of MWCNT results in the formation of a reddish-black precipitate. Microscopic inspection of this precipitate under epifluorescence visualises small fluorescent puncta with the characteristic shape of MWCNTs. This observation concurs with previous reports showing binding of DiI to CNTs with high affinity [25]. MWCNT/DiI was injected as a colloidal suspension in 10% DMSO/water (see Experimental Section). As control we injected 100 µg/ml DiI in 50% DMSO to visualise the spread and accumulation of dye not bound to MWCNTs (Figure 1B). In contrast to 1 mg/ml DiI in DMSO, which diffused easily throughout the embryo labelling internal membranes (Figure 1B), injected MWCNTs formed small puncta, which only dispersed throughout four segments in the ventral half of the embryo (Figure 1C), equalling about 6% of total embryonic volume. The emission of unbound dye is no longer detectable after three nuclear divisions (data not shown) but the dye labelled MWCNT puncta can be followed throughout embryogenesis (Figure 1D–G) indicating that the dye remains bound to MWCNTs and does not diffuse away.

Injected MWCNTs stay mainly in the cytoplasm and rarely associate with microtubules (Movie S1). Even if MWCNTs collide with microtubules, microtubule based spindle formation proceeds normally (arrows, Movie S1). The presence of labelled or unlabelled MWCNTs does not interfere with the signalling process controlling the ingression of membranes during cellularisation [24]. In addition, MWCNT accumulation does not impede gastrulation (Movie S2), a highly coordinated movement of epithelial cells enforced by the apical constriction of actin [26]. Hence, MWCNT do not interfere with the formation and contraction of the two major motile fibre systems, microtubules and actin.

After cellularisation, MWCNTs are incorporated into ectodermal stem cells, which give rise to the epidermis (skin, Figure 1D, E) and into neural stem cells giving rise to neurons and glial cells, which become part of the central nervous system (CNS). Interestingly the presence of MWCNTs does not interfere with the extension and direction of neuronal axons (Figure 1F). At the end of embryogenesis, MWCNT have been incorporated into the CNS without causing any visible axonal damage (Figure 1G).

*Drosophila* embryos possess an innate immune system with a humoral component consisting of secreted anti-bacterial and anti-fungal peptides, and a cellular component made up of haematocytes that recognize and engulf foreign bodies and cell fragments [27]. Due to their increased size and their high motility, haematocytes can be easily detected and followed in the living embryo. During embryogenesis not all injected MWCNTs become incorporated in cells. Some MWCNT stay extracellular and together with MWCNT released from dying cells (see below) accumulate in older embryos. We recorded the behaviour of haematocytes around MWCNT deposits and surprisingly found that haematocytes do not specifically target these deposits (Movie S3). Although we detected some haematocytes with engulfed MWCNTs attached to cell debris (Movie S4), MWCNTs only seem to invoke a weak immune response in *Drosophila* embryos.

Finally, we tested the overall viability of embryos injected with MWCNTs and controls. At the end of development, healthy embryos will hatch as larvae. Hatching rate can therefore be used as readout for damage caused during embryogenesis. Hatching rate is never 100% because the preparation of embryos for injections always interferes with larval viability. Yet, as expected from our previous results the injection of either unlabelled or labelled MWCNTs did not reduce hatching rate as compared to water-injected controls (Figure 1H).

We conclude that the presence of intracellular MWCNTs has no observable effect on cell motility, cell communication, tissue and organ formation, phagocytosis or general viability of developing *Drosophila* embryos.

### MWCNTs do not arrest DNA replication

**Figure 2:**
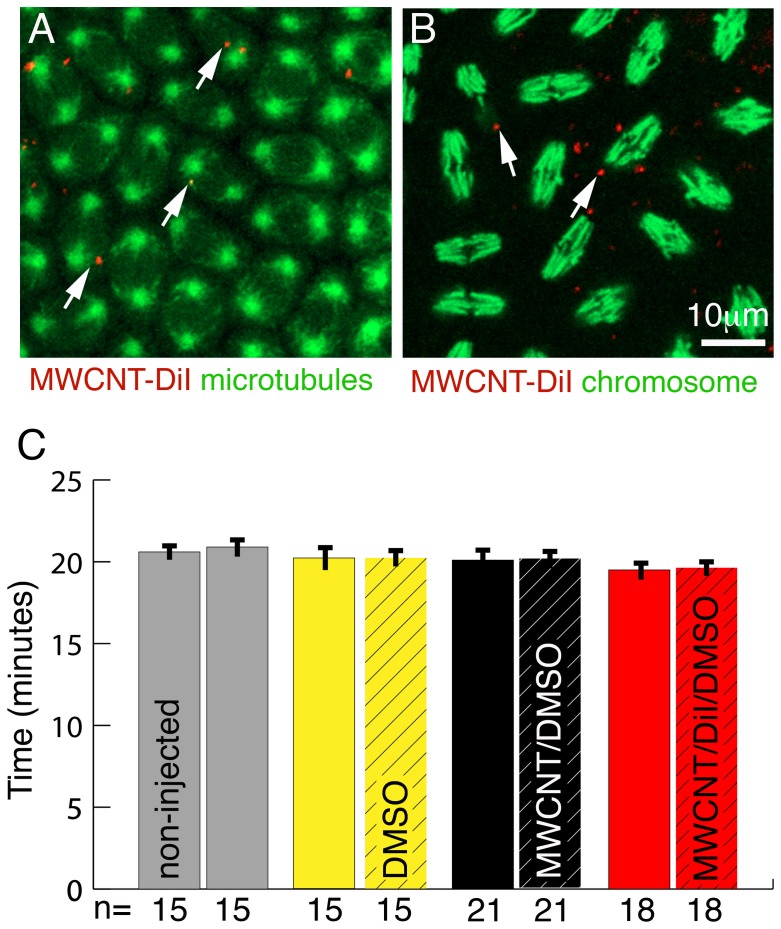
MWCNTs do not interfere with cell division. Ventral views; Anterior is up. Bar equals 10 µm in A, B. (A) Single live confocal section through the syncytial blastoderm during a division wave. Microtubules are labelled with GFP (green, Jupiter-GFP). All nuclei are in the same stage (metaphase) of the cell cycle even if MWCNTs (red, arrows) are present. (B) Single live confocal section through an embryo with YFP labelled histones marking chromosomes (green). Regardless of the presence or absence of MWCNTs (red, arrows), all nuclei are in the same stage of division (anaphase). (C) The rate of nuclear divisions is not affected by the presence of MWCNTs. Division times for three nuclei per embryo half were recorded and division times between non-injected (non-hatched) and injected (hatched) halves compared. For non-injected embryos (grey) division times of three randomly chosen nuclei for each half were recorded. We detect no differences in the division times between non-injected and injected halves nor between embryos injected with 10% DMSO (yellow, solvent control), 1 mg/ml MWCNT in 10% DMSO/water (black, MWCNT/DMSO) and 1 mg/ml MWCNT in 10% DMSO labelled with DiI (red, MWCNT/DiI/DMSO). Y-axis, division time in minutes; X-axis, Number of nuclei analysed (n).

The synchronised nuclear divisions in the syncytial blastoderm of *Drosophila* embryos offers the unique opportunity to check if the presence of MWCNTs interferes with DNA replication, and thereby slowing down or speeding up divisions. In addition, failure in DNA replication or chromosomal separation can easily be detected because these nuclei fall out of the embryonic surface into the yolk [28]. We injected MWCNTs and control vehicles into the syncytial blastoderm and recorded the last four synchronous nuclear divisions along the surface of the embryo (Movie S5, S6, S7). Nuclei surrounded by MWCNTs divided at the same time as adjacent MWCNT-free nuclei (Figure 2A, B). We did not detect any difference in the division cycles between control and MWCNT injected embryos or between injected and non-injected embryo halves (Figure 2C). We also did not observe any increase in nuclear fallout between embryos injected with MWCNTs or controls (data not shown). We conclude that MWCNTs do not interfere with DNA replication or chromosome separation. Interestingly, we never detected a nuclear incorporation of MWCNTs although the breakdown of the nuclear membrane during nuclear divisions would permit MWCNT entry. This argues for an yet to be described mechanism, which actively prevents the nuclear entry of MWCNTs.

### MWCNTs do not induce DNA double-strand breaks

**Figure 3:**
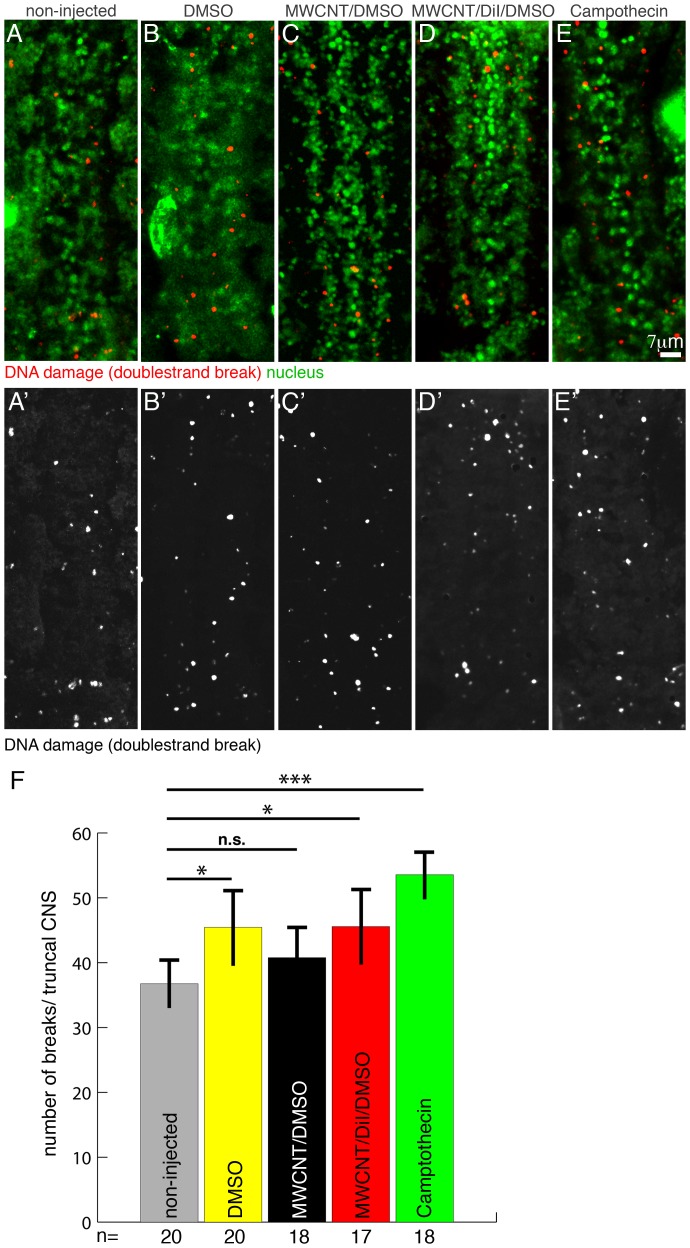
MWCNTs do not cause a significant increase in DNA double-strand breaks. Ventral views; Anterior is up. Bar equals 7 µm in A–E′. Embryos were allowed to develop for 8 h after MWCNTs injection before fixation and immunohistochemistry. (A–E′) Immunostainings of double-strand breaks (red; anti-γH2Av) and nuclei (green; YFP-HistoneH2a, anti-GFP) in non-injected embryos and embryos injected with 10% DMSO (DMS0), 1 mg/ml MWCNT in 10% DMSO/water (MWCNT/DMSO), 1 mg/ml MWCNT in 10% DMSO labelled with DiI (MWCNT/DiI/DMSO) and Camptothecin (media indicated on top of each image). A′–E′ shows red channel only in grey. (F) The presence of MWCNT does not increase the frequency of DNA double-strand breaks. To assess the number of double-strand breaks we counted all γH2Av puncta located in nuclei of the truncal CNS (1^st^ thoracic to 7^th^ abdominal segment). Camptothecin, an agent known to cause double-strand breaks, served as a positive control. The only highly significant increase in the frequency of breaks can be observed between non-injected (grey) and Camptothecin (green) injected embryos. We detect a significant increase between non-injected embryos and embryos injected with 10% DMSO (yellow, DMSO) or 1 mg/ml MWCNT labelled with DiI in 10% DMSO (red, MWCNT/DiI/DMSO). Yet, injection of 1 mg/ml MWCNT in 10% DMSO/water (black, MWCNT/DMSO) does not cause more double-strand breaks (pairwise comparison with non-injected embryos; t-Test). n.s., non significant; *, significant (p<0.05); ***, highly significant (p<0.001). Y-axis, number of breaks/truncal CNS; X-axis, Number of embryos analysed (n).

A complex DNA repair system in eukaryotes ensures that during cell divisons chromosomes are segregated faithfully, and the stability of the genome and its information is preserved [29]. Endogenous recombinations and a multitude of external agents can cause genome instability resulting in mutations, genome rearrangements, chromosome fragmentation and chromosome loss. Genome instability is the major cause of cell death, aging and cancer [22]. A major trigger of genome instability is the unfaithful repair of DNA double-strand breaks. DNA double-strand breaks are marked for repair by a phosphorylated variant of the DNA binding molecule Histone2, γH2Av [30]. We used an antibody [31] to detect γH2Av after injection of MWCNTs (Figure 3). We injected labelled and unlabelled MWCNTs into the syncytial blastoderm along with vehicle controls (10% DMSO) and a positive control, Camptothecin, a substance known to cause DNA double-strand breaks [32]. Nine hours after injection the embryos were fixed and immunostained for γH2Av. The nuclei of the injected embryos were marked by the expression of YFP-Histone *2A (Gal4^V2h^/UAS::YFP::Histone 2A)*. We scored all γH2Av positive signal spots colocalising with the nucleus as double-strand breaks (Figure 3A–E′). Only the 10 truncal segments of the nervous system which originate from the injection site were analysed. As expected, we detected a highly significant (p<0.001) increase in the number of DNA double-strand breaks between non-injected embryos and Camptothecin injected embryos (Figure 3F). We also detected a significant (P<0.05) increase of double-strand breaks in embryos injected with 10% DMSO and DiI labelled MWCNTs. Yet, these increases seem to be due to DiI or DMSO since compared to non-injected embryos, the injection of unlabelled MWCNTs does not increase the frequency of DNA double-strand breaks (Figure 3F). We conclude that the intracellular presence of MWCNT does not cause DNA double-strand breaks and hence does not significantly increase genotoxic stress.

### MWCNTs increase cell death of ectodermal stem cells

Injections of MWCNTs into the ventral region of *Drosophila* embryos allow following the influence of CNTs on survival, division pattern and progeny differentiation of two kinds of stem cells. Ectodermal stem cells, which give rise to the outer body cover, the larval epidermis and some cells of the sensory nervous system, and neural stem cells, which generate the neurons and glial cells in the ventral part of the central nervous system, the equivalent to the spinal cord in vertebrates [33], [34].

**Figure 4:**
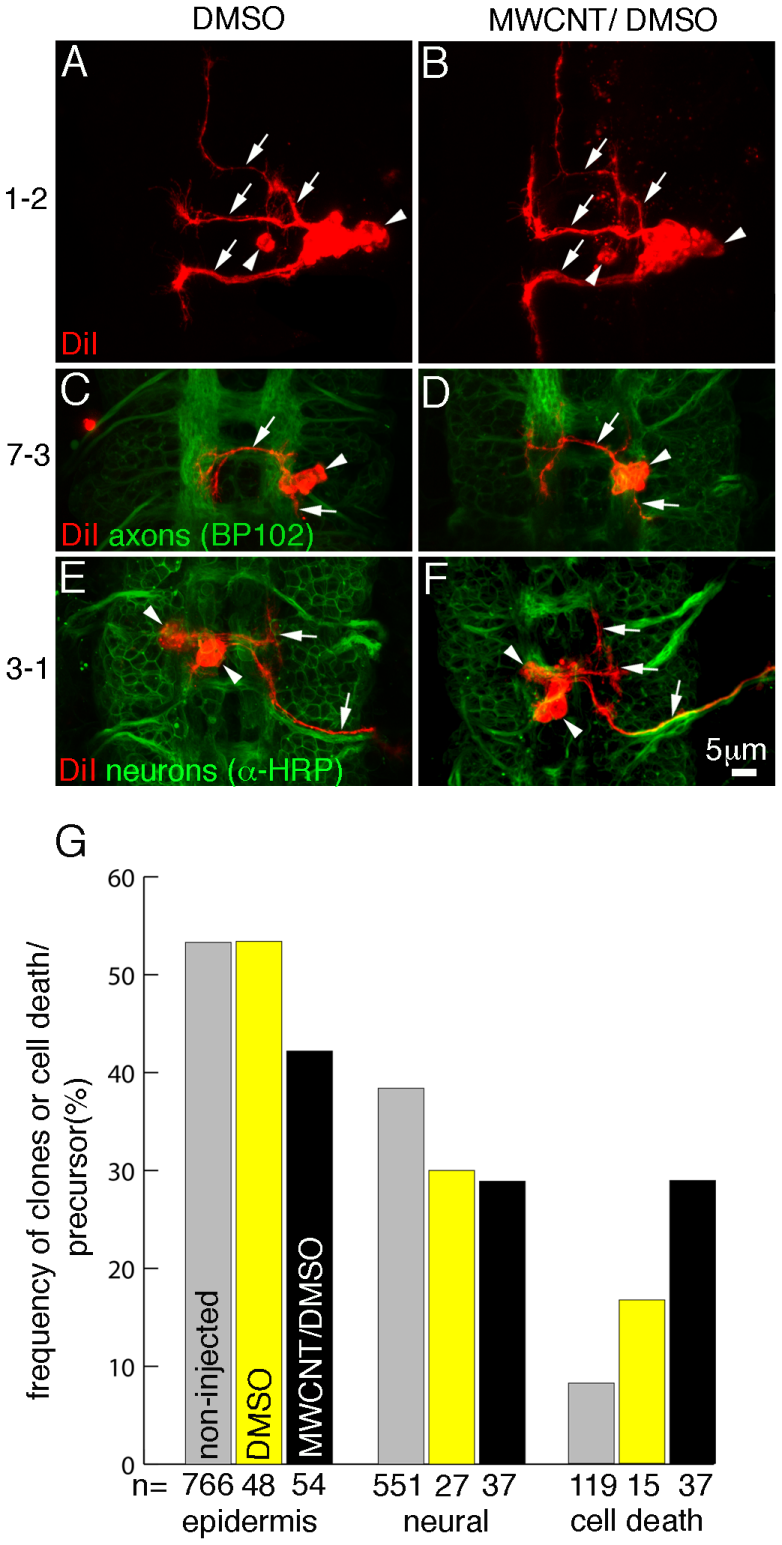
MWCNTs do not interfere with differentiation but increase cell death of ectodermal stem cells. Ventral views; Anterior is up. Bar equals 5 µm in A–F. Single neural stem cells were labelled with DiI (red) in embryos injected with 10% DMSO (DMSO; A, C, E) or with 1 mg/ml MWCNT in 10% DMSO/water (MWCNT/DMSO; B, D, F). The identification of the stem cell (1-2, 7-3, 3-1) is given on the left. (A–F) MWCNT does not interfere with neuronal differentiation. Labelled stem cells were allowed to divide and the progeny were examined for axonal extension (arrow), cell body position (arrowhead) and cell number. To examine the overall development of the CNS either all axons (anti-BP102; C, D) or all neuronal membranes, axons and cell body (anti-HRP; E, F) were immunostained. We do not detect any differences between stem cell progeny in non-injected embryos [34], DMSO or MWCNT/DMSO injected embryos. Note that the difference in branching pattern of 3-1 progeny (E, F) is caused by their different anterior-posterior location [34] and not MWCNT injections. (G) Frequency and kind of progeny obtained from labelled embryonic stem cells. Compared to non-injected embryos (grey), the injection of DMSO (yellow) results in an increase of cell death of neural stem cells. Injection of MWCNT/DMSO (black) affects the survival of epidermal as well as neural stem cells resulting in a significant (p<0.05) increase in cell death. The cell death increase observed in the case of neural stem cells is not higher than upon injection of DMSO only. Y-axis, frequency in % of epidermal (epidermis; column 1–3) or neural progeny (neural; column 4–6) or cell death (column 7–9) arisen from labelled stem cells; X-axis, total number of cells labelled (n). Numbers for non-injected embryo are taken from Bossing et al., 1996.

We injected MWCNTs and vehicle control (10%DMSO in water) into the syncytial blastoderm. 1.5 h after injection we labelled three to six cells at the injection site with the lipophilic dye DiI [21]. The dye is transferred from the stem cell to the progeny and labels the cell membrane of all progeny (Figure 4). We allowed the embryos to complete their development before we processed them for immunostaining and recorded the progeny. Consistent with our previous results (Figure 1), immunostaining against axonal or neuronal surface proteins did not show any gross morphological disturbances of the nervous sytem. This result is supported by the observed normal differentiation of epidermal and neural progeny derived from the labelled stem cells (Figure 4A–F). Ectodermal stem cells in non-injected embryos can give rise to 2–12 progeny with an average of 6.07 (+/−2.82, n = 202). In embryos injected with 10% DMSO progeny number varies between 2–11, average 6.04 (+/−2.56, n = 25) and injection of MWCNTs result in 2–12 daughter cells/ectodermal precursor, average 6.27 (+/−2.78, n = 48). The number of progeny derived from neural stem cells is stem cell specific [34] and we did not detect any differences between identical stem cells labelled in non-injected, 10% DMSO injected, and MWCNT injected embryos. We conclude that the presence of MWCNTs does not affect the number of progeny i.e. the division pattern of precursors cells.

If labelled stem cells or progeny die we detect at the end of embryogenesis labelled cell debris in haematocytes accompanied by a reduced number or a complete loss of progeny. In 10% DMSO injected embryos we noticed an increased cell death of neural stem cells but not ectodermal stem cells. In MWCNT injected embryos, cell death of neural stem cells is as high as in 10% DMSO injected controls, arguing that DMSO is the insulting agent. In contrast, cell death of ectodermal stem cells in MWCNT injected embryos was higher than in embryos injected with 10% DMSO alone (Figure 4G). We conclude that the intracellular presence of MWCNTs reduces viability of distinct types of stem cells.

## Discussion

The increased production and application of CNTs makes the testing of their environmental and health impact an urgent necessity. The ingestion of CNTs by model organisms such as mice, *Drosophila*, *C. elegans* and zebrafish is a convenient way to study the impact of CNTs released into the environment (e.g. [12], [15], [35], [36]). Yet, ingested CNTs mainly reside in the external digestive system and their cellular uptake is limited [37]. Future medical applications of CNTs will cause permanent exposure of internal organs, e.g. by the implantation of CNT coated stents or scaffolds. Wear of these scaffolds will release CNT particles and promote the subsequent cellular uptake by phagocytic cells of the immune and digestive system. In the case of drug delivery systems to combat cancer or promote stem cell proliferation, uptake into dividing cells is the ultimate objective [5]. We show that injection of CNTs into early *Drosophila* embryos can serve as a cost effective, convenient and informative testing system to study the biological effects of cellular uptake of MWCNTs.

We labelled MWCNTs by incubation with the lipophilic dye DiI dissolved in DMSO. In agreement with previous results [25], microscopical inspection under epifluorescence of the precipitate after several washes with DMSO confirms the binding of the dye to MWCNTs. Injected DiI dissolved in DMSO spreads through cellular membranes and rapidly becomes diluted during cell divisions. In contrast, injected MWCNT/DiI/DMSO solutions do not show any diffusion of the dye into membranes but form puncta inside the cytoplasm. These puncta can be visualised throughout embryogenesis, indicating that the dye stays bound to MWCNTs after injection into embryos.

We show that injection of labelled and unlabelled MWCNTs into the syncytial blastoderm of the embryo does not interfere with nuclear divisions and results in the cellular uptake of MWCNTs when nuclei become enclosed by ingrowing cell membranes. Using transgenic embryos with cellular substructures labelled by fluorescent proteins, we detect no accumulation of intracellular DiI labelled MWCNTs along microtubules, cell membranes or inside nuclei. Intracellular MWCNTs are dispersed throughout the cytoplasm and randomly distributed during cell divisions.

Organ formation during embryogenesis relies on cell communication achieved by an intricate network of molecular signalling pathways [38]. The extracellular and intracellular presence of MWCNT seems not to disrupt organ formation and hence does not reduce embryonic viability. Our single cell analysis of the developing CNS, the most complex organ, demonstrates that MWCNTs do not alter stem cell divisions or neuronal and glial differentiation. Surprisingly, axon outgrowth and guidance, processes controlled by various signalling pathways [39], [40], proceed normally in the presence of MWCNTs, which even become incorporated into the mature larval CNS without any visible disturbances.

Two types of stem cells originate from the injected embryonic ventral region, ectodermal stem cells, giving rise to the epidermis and sensory nervous system of the mature larvae, and neural stem cells giving rise to neurons and glia cells. All the progeny of both stem cell types have been identified [34], [41]. The development and division pattern of these stem cells is tightly controlled. For example, following the loss of function of tumour suppressor genes, an increased number of undifferentiated progeny from neural stem cells is observed [42]. Yet, the presence of MWCNTs does not interfere with cell number or differentiation of neural progeny. In addition, MWCNT injections do not increase the frequency of DNA double-strand breaks, which are a major cause of genotoxic stress and carcinogenesis [22].

Surprisingly, we detect that the type of stem cell defines the risk of damage caused by the cellular uptake of MWCNTs. Ectodermal stem cells exhibit increased cell death but not neural stem cells. Both stem cell types originate from the same cell layer by cell-cell interactions from a cluster of about 4–6 cells which are morphologically and genetically equivalent [43], [44]. One cell of this cluster will become a neural stem cell and the remaining cells adopt the ectodermal stem cell fate. At the time of the first wave of selection, shortly after gastrulation, we do not detect an accumulation of MWCNTs in only one cell but a spread over adjacent cells (Movie S2). It is unlikely that minor differences in MWCNT intracellular load increases the possibility to become an ectodermal stem cell and therefore increases the number of ectodermal stem cell death. Such an imbalance in cell fate would also lead to a reduced number of labelled neural stem cells, which we do not observe. Immediately after their selection both stem cell types become different from each other not only in their mode and number of divisions but also in their molecular identity. Ectodermal stem cells stay in the superficial tissue they originated in and divide symmetrically and equally, whereas neural stem cells delaminate to form a new tissue below the ectodermal layer and divide asymmetrically and unequally [33]. Ectodermal stem cells give rise to a maximum of 12 progeny but neural stem cells can give rise to up to 40 progeny [34]. If number of divisions and hence dilution of the MWCNT load would be crucial for stem cell survival, we would expect that early born progeny which inherit a larger load of MWCNT are less viable than later born progeny resulting in a reduction of the average number of progeny per stem cell. However, the average number of progeny per stem cell in injected and non-injected embryos is not different. Hence, the reduced number of divisions in ectodermal stem cells seems not to be the cause for the increased cell death. Future studies using for example current sequencing techniques allowing the rapid identification of transcripts differentially expressed in both stem cell types, will aid the identification of the pathways responsible for the increased susceptibility to MWCNT caused damage in ectodermal stem cells versus neural stem cells.

We demonstrate that *Drosophila* embryos can be used as a model to test the toxicity of CNTs in unprecedented detail. *Drosophila* embryos are an excellent system to study developmental toxicity because of easy and inexpensive maintenance, ready availability and the possibility of automating embryo injections. Can results obtained from injections into embryos be used to evaluate toxicity of CNTs for other animals and even humans? *Drosophila* has a long-standing history as genetic model for human diseases [45]. All signalling pathways and most of the organs in humans have analogous pathways or organs in *Drosophila*, which is also expressed in the high conservation rate of 55% of all genes between the *Drosophila* and human genomes [45], [46]. Basic cell biological aspects such as cell division including oncogenesis, transcriptional regulation, membrane biology, DNA integrity and repair, cell movement and tissue formation are conserved all over the animal kingdom, and often have been first studied in *Drosophila*. These arguments underline the strength of *Drosophila* embryos as a novel model for future studies of CNT developmental toxicity.

## Materials and Methods

### Fly strains

We used the GAL4 driver, GAL4^V2h^ [47], the protein trap Jupiter-GFP [23] and UAS-YFP::HistoneH2 [48].

### Carbon nanotube labelling and injections

MWCNT were supplied by NanoAmor Europe (Cat.No. 1233YJ, ID: 5–15 nm, OD: 50–80 nm, Length 10–20 µm; analysis by Energy Dispersive X-ray Spectroscopy: C: 97.37%, Cl: 0.2%, Fe: 0.5%, Ni: 1.86%, S: 0.02%). We dissolved 10 mg MWCNTs in 1 ml 100% DMSO (Sigma, Cat. No. D8418, 99.9% pure, impurities: <0.1 water, <0.001 meq/g titratable acid) with or without the addition of 1 mg of the lipophilic dye, DiI (Cat. No. D-282, Invitrogen). Both solutions were vortexed on high speed for about 2 min to coat MWCNTs with DMSO or DMSO/DiI. DiI labelled MWCNTs were washed five times with 100% DMSO to remove excess, unbound dye. After final wash, MWCNTs were spun down with a benchtop centrifuge at 6000 rpm and checked for fluorescence [20× objective, Numerical aperture (N.A.): 0.75, 10× ocular, excitation: 510–560 nm, dichroic: 565 nm, Barrier: 590 nm], Stocks were kept at 4°C in the dark. DiI label of MWCNTs is stable for about 6 weeks. For injections we warmed the stock solution and de-ionised water at 65°C for 10 min, added 10 ul of the stock solution into 90 ul of water and vortexed the solution for 5 min at medium speed. After vortexing, MWCNTs form a transient colloidal dispersion, which has to be injected immediately. Before injection, CNTs in the solution was examined for dispersion and epifluorescence under 200× magnification (Nikon TE 300, 20× objective, N.A. 0.75, 10× ocular). We injected MWCNTs using borosilicate capillaries (Harvard Apparatus; ID: 0.75 mm, OD: 1.5 mm, no inner filament) pulled on a Sutter P-97 micropipette puller and with a tip bevelled to an angle of 25° using a Bachofer Microtip Grinder. Embryos were de-chorionated manually on double-sided tape, aligned on a block of 2.5% Agar and transferred with their ventral side down onto a coverslip (24 mm×60 mm) of which the middle was coated with glue and to which a plastic frame was attached (cut out from self adhesive book binding foil, ID: 16 mm×16 mm, OD: 22 mm×22 mm). The coverslip was supported with a microscope slide, embryos were desicated at room temperature for 5–10 min (depending on humidity) and covered with halocarbon oil (Voltalef 10 s) to stop further evaporation. Injection of embryos was controlled with 100× magnification (10× objective, N.A.0.45, 10× ocular), which allows monitoring of dispersion of MWCNTs in the capillary. On average about 20 pl of the 1 mg/ml solution, equalling about 4% of total embryo volume, was injected equalling 5 pg of MWCNTs/embryo. MWCNTs only showed a limited diffusion covering about 6% of total embryo volume. Embryos injected as control with water, 10% DMSO, unlabelled MWCNTs or with Camptothecin were treated the same way.

### Immunohistochemistry

The following antibodies were used BP102 (mouse, 1∶1000; kindly provided by Professor Nipam Patel), anti- γH2Av (1∶1000; kindly provided by Kim McKim) and anti-HRP coupled to FITC (goat, 1∶500, MPbio). Embryos were stained as fillets as previously described [34]. All antibodies are used for routine stains in *Drosophila* embryonic research and their staining pattern has been well described [49] [31]

### Time lapse, Confocal and Image processing

Fixed and live samples were recorded using a Zeiss LSM710. For time lapse recording of living embryos, recording started 15 min after injection and intervals were set to 30 sec. A maximum of three Z-levels covering 2 µm were scanned bi-directionally for each time point. Movies were generated using the ZEN software (Zeiss) and Image J. Movies and Images were assembled and labelled using Photoshop CS6.

## Supporting Information

Movie S1 **MWCNT do not interfere with microtubule formation.** 1 mg/ml MWCNT/DiI/DMSO (red, arrows) was injected into the syncytial blastoderm. Movie covers 60 min after injection with a frame taken every 30 sec. Bar, 10 µm. Microtubules are visualised by the expression of Jupiter-GFP, a microtubule binding protein. MWCNTs mainly accumulate in the cytoplasm but can randomly hit microtubules (arrows). These interactions are transient and do not interfere with the formation of the mitotic spindle, a structure made of microtubules and essential for the equal separation of DNA. In total 10 embryos were injected and recorded.
(MOV)

Movie S2 **MWCNT do not interfere with gastrulation, a major cell movement.** 1 mg/ml MWCNT/DiI/DMSO (red, arrows) was injected into the syncytial blastoderm. MWCNTs incorporated into the cytoplasm during cell formation. Cells are outlined by GFP tagged microtubules (green, Jupiter-GFP). Movie covers 70 min of development with a frame taken every 30 seconds. Bar, 10 µm. Apical cell constrictions form the ventral furrow (arrowhead) through which the ventral tissue moves into the embryo. Note that cell movements at the side of MWCNT deposition (red, arrows) proceed normally. In total, 6 embryos were injected and recorded.
(MOV)

Movie S3 **MWCNTs are not readily engulfed by phagocytic cells of the immune system.** MWCNT/DiI/DMSO (red, arrows) were injected into the syncytial blastoderm and mostly incorporated into the cytoplasm of the newly formed cells. Cells are outlined by GFP tagged microtubules (green, Jupiter-GFP). Movie covers 103 min with a frame taken every 1 min. Bar, 10 µm. Haematocytes, phagocytic and migratory cells of the innate immune system (three arrowheads at beginning of movie), become active about 600 min after injection (720 min after fertilisation), clearing cell fragments and foreign substances by phagocytosis. Uptake of debris involves filopodia, thin cellular extensions which are highly mobile. At the end of the movie, labelled MWCNTs (red, arrow) are located close to a haematocyte. The haematocyte extends a filopodium (arrowhead) but this filopodium although directed towards the MWCNTs does not pull the MWCNT into the cell, showing that extracellular MWCNT are not readily recognized by Haematocytes. In total, 5 embryos were injected and recorded.
(MOV)

Movie S4 **Macrophages engulf MWCNTs attached to cell debris.** Same embryo as in movie 2, 840 min after injection (960 min after fertilisation). At the end of embryogenesis, macrophages (arrowhead) have engulfed debris of dead cells (bright green spots) and also show an accumulation of MWCNTs (red, arrow). Movie covers 120 min with a frame taken every 1 min). In total, 5 embryos were injected and recorded.
(MOV)

Movie S5 **Injection of 10% DMSO does not disrupt nuclear divisions in early embryos.** 10% DMSO in water was injected into the syncytial blastoderm of transgenic embryos with all nuclei labelled with Histone-YFP (green, *GAL4^V2h^/UAS-YFP::HistoneH2*). Movie starts 15 min after injection and covers 2 h of embryogenesis. Frame recorded every 30 sec. Bar, 10 µm. Nuclei on the injected right side continue to divide synchronously which shows that DMSO injections do not interfere with DNA replication or the division machinery. 5 embryos were injected and recorded. See Figure 2C.
(MOV)

Movie S6 **Injection of MWCNT/DMSO does not disrupt nuclear divisions in early embryos.** MWCNT (1 mg/ml) in 10% DMSO and water was injected into the syncytial blastoderm of transgenic embryos with all nuclei labelled with Histone-YFP (green, *GAL4^V2h^/UAS-YFP::HistoneH2*). Movie starts 15 min after injection and covers 2 h of embryogenesis. Frame recorded every 30 sec. Bar, 10 µm. Nuclei on the injected right side continue to divide synchronously which shows that MWCNT do not disrupt DNA replication or the division machinery. 7 embryos were injected and recorded. See Figure 2C.
(MOV)

Movie S7 **Injection of MWCNT/DiI/DMSO does not interfere with nuclear divisions in early embryos.** MWCNT (1 mg/ml)/DiI/DMSO in water (red) was injected into the syncytial blastoderm of transgenic embryos with all nuclei labelled with Histone-YFP (green, *V2hGAL4/UAS-YFP::HistoneH2*). Movie starts 15 min after injection and covers 90 min of embryogenesis. Frame recorded every 30 sec. Bar, 10 µm. Nuclei on the injected right side continue to divide synchronously. Note that MWCNT do not become incorporated into the nuclei. 6 embryos were injected and recorded.
(MOV)
